# Neutrophil-to-lymphocyte ratio and platelet-to-lymphocyte ratio as potential predictors of nosocomial infection in patients undergoing veno-arterial extracorporeal membrane oxygenation: A cohort study

**DOI:** 10.1371/journal.pone.0325316

**Published:** 2025-06-03

**Authors:** Zishu Xu, Lu Qi, Shiqiong Su, Zhijing Xu, Yuan Geng, Yangang Shi, Congmei Wang, Jie Wu, Ruifang Liu

**Affiliations:** Department of Intensive Care Medicine, The Third People’s Hospital of Henan Province, Zhengzhou, China; Government Villupuram Medical College and Hospital, INDIA

## Abstract

**Objective:**

The aim of this study was to evaluate and compare the predictive value of the neutrophil-to-lymphocyte ratio (NLR) and platelet-to-lymphocyte ratio (PLR) for nosocomial infection in patients undergoing veno-arterial extracorporeal membrane oxygenation (VA-ECMO).

**Methods:**

In this retrospective cohort study, we assessed 95 patients who underwent VA-ECMO between 2018 and 2023 at the Third People’s Hospital of Henan Province. The association between NLR, PLR and nosocomial infection in patients undergoing VA-ECMO was tested using logistic regression model.

**Results:**

Among 95 VA-ECMO patients, 30 were found to have developed nosocomial infection (infection rate 31.58%). Patients with higher levels of NLR and PLR showed higher rates of nosocomial infections (p < 0.05). Higher NLR were associated with an increased risk of nosocomial infections in patients undergoing VA-ECMO (odds ratio [OR], 4.858; 95% confidence interval [95% CI], 1.864–12.663) (P = 0.001), after adjusting for sex, age, hemoglobin, albumin, and duration of VA-ECMO treatment. In reference to the first tertile of NLR, ORs were 6.931 (95% CI, 1.496–32.118) for the second tertile, 8.898 (95% CI, 1.943–40.751) for the third tertile. PLR was a risk factor for nosocomial infections in patients undergoing VA-ECMO (OR 5.478; 95%CI 2.117–14.176) after adjusting for traditional risk factors (P < 0.001). ROC curve analysis showed that the area under the curve (AUC) of NLR and PLR to predict nosocomial infections in patients treated with VA-ECMO were 0.710 and 0.763, respectively.

**Conclusions:**

High NLR and PLR were associated with an increased risk of nosocomial infection in patients treated with VA-ECMO.

## Introduction

VA-ECMO provides temporary mechanical circulatory support and extracorporeal gas exchange at the same time for severe cardiopulmonary failure [1,2]. The utilization of VA-ECMO has witnessed a significant surge in recent years [3,4]. The in-hospital mortality rate for patients undergoing VA-ECMO, however, reached as high as 57.2% [5]. Various complications can occur during VA-ECMO. Nosocomial infection following ECMO, as one of its notable complications, can significantly increase the mortality of patients [6]. A meta-analysis definitively demonstrated that nosocomial infections resulted in a 32% increase in in-hospital mortality [7]. Therefore, infection prevention and early detection strategies are critical aspects of ECMO management [8].

Main sources of ECMO-related nosocomial infection include bloodstream infections (BSIs), urinary tract infections (UTIs), surgical site infections (SSIs), and ventilator-associated pneumonia (VAP) [9]. Among them, VAP and BSI were the most common nosocomial infection sources in patients on ECMO, accounting for 33% and 15%, respectively [10]. Common pathogens include Staphylococcus Aureus (often methicillin resistant), non-lactose fermenting gram-negative bacilli and Candida [11].

The initiation of ECMO elicits an immediate and intricate inflammatory response, akin to the systemic inflammatory response syndrome (SIRS) [12]. In ECMO applications, the exposure of blood to abiotic surfaces, blood shear stress, and air-blood interfaces can initiate and enhance a systemic inflammatory response [13]. ECMO may trigger an inflammatory process, which has been correlated with increased rates of morbidity and mortality [14]. The existing biomarkers for patients with suspected infections have limitations in terms of sensitivity and specificity, and blood cultures are slow to identify organisms, with the possibility of false negatives. NLR and PLR are new white blood-cell-based inflammatory markers which are readily available [15]. Some studies have shown that NLR and PLR are important markers of inflammation, which can reflect the infection status and the degree of inflammation [16,17]. Studies revealed that NLR and PLR serve as reliable indicators for assessing the prognosis of a wide range of diseases [18–20]. However, the significance of NLR and PLR in nosocomial infection after VA-ECMO remains unclear. In this study, we aimed to systematically evaluate and compare the predictive capacities of NLR and PLR for nosocomial infections in VA-ECMO patients by: (1) conducting head-to-head comparisons of their diagnostic performance (AUC, sensitivity, specificity); (2) assessing their independent predictive values through multivariable regression adjusted for clinical confounders; and (3) identifying optimal biomarker thresholds to guide clinical decision-making.

## Materials and methods

### Study design and participants

This retrospective study included adult patients who received VA-ECMO support treatment between 15/12/2018 and 20/08/2023 at the Department of Intensive Care Medicine of the Third People’s Hospital of Henan Province and the data were accessed for research purposes from 10/05/2024–26/05/2024. Patients with any infection before receiving ECMO support, age ≤ 18 years, use of veno-venous or veno-arterial venous ECMO, ECMO support ≤48 hours, advanced malignancies (due to inherently altered NLR/PLR baselines and limited ECMO survival benefit), hematological diseases affecting neutrophil, lymphocyte, or platelet counts (e.g., acute/chronic leukemia, myelodysplastic syndrome, etc.), and incomplete clinical data were excluded from the analysis. Infections before receiving ECMO support were ruled out based primarily on the patient’s history, clinical presentation, analysis of antimicrobial therapy records, laboratory tests (e.g., blood cultures, C-reactive protein, procalcitonin, white blood cell count), and imaging tests (e.g., CT scans or chest X-ray).

### Definition of nosocomial infection related to ECMO

Nosocomial infections related to ECMO were defined as a case with confirmed organisms from one or more blood, respiratory, or urinary cultures during the period 24 hours after the initiation of ECMO to 48 hours after its discontinuation [21], and based on the Diagnostic Criteria for Nosocomial Infections (Proposed) issued by the National Health Commission of the People’s Republic of China in 2001, which referenced the definition of Nosocomial infection by the American Centers for Disease Control and Prevention in 1988 [22]. Microbiological isolations were correlated with clinical symptoms and typical inflammatory characteristics in blood samples and radiographic findings.

### Data collection

Clinical data, including age, sex, underlying disease, laboratory indicators, duration of ECMO support were collected. All laboratory indicators were collected within 24h after the initiation of VA-ECMO. The first measurement after initiation was selected if multiple values were available. White blood cells and platelets were measured using a fully automated modular blood fluid analyzer (Sysmex XN-10[B4], Sysmex Corporation, Kobe, Japan). NLR was calculated by dividing the absolute neutrophil count by the absolute lymphocyte count. PLR was calculated in a similar manner, with the platelet count divided by the absolute lymphocyte count.

The Samples collected from patients with nosocomial infection after ECMO, included bronchoalveolar fluid, sputum, blood, midstream urine, feces and swab from the infection site. The isolated colonies were identified using the fully automated BD Phoenix™ M5 system (Becton Dickinson, USA). Quality control strains, including Acinetobacter baumannii (ATCC 19606) and Klebsiella pneumoniae (ATCC 700603), were obtained from the National Center for Clinical Laboratories.

### Statistical analysis

Continuous variables, NLR and PLR, were categorized into tertiles (low, medium, high) based on their distribution within the study cohort (n = 95). The tertile cut-offs were determined using percentile ranks: the first tertile (low) was defined as values below the 33.3 percentile, the second tertile (medium) as values between the 33.3 and 66.6 percentiles, and the third tertile (high) as values above the 66.6 percentile. Normally distributed variables were expressed as mean ± standard deviation (SDs) and compared using the t-test; non-normally distributed variables were presented as median and interquartile range (IQR) and compared using the Kruskal-Wallis test. Categorical data were presented as percentages or frequencies and were tested using the chi-square test. The relationship between NLR, PLR and the risk of nosocomial infection in patients treated with VA-ECMO were estimated using logistic regression models. Traditional risk factors (age, sex, proteinuria, hemoglobin, albumin, duration of VA-ECMO treatment) were adjusted in multivariable logistic regression models. The Receiver-operating characteristic (ROC) curve analysis was used to analyze the predictive value of NLR and PLR for nosocomial infection in patients treated with VA-ECMO. The ROC curve was drawn to analyze sensitivity, specificity, cut-off values, the Youden index and the area under the ROC curve. Statistical analysis was performed using SPSS (version 26.0; SPSS, Chicago, IL, USA). Statistical significance was set at p < 0.05.

### Ethics statement

The study was conducted in accordance with the principles of the Declaration of Helsinki and received ethical approval from the Ethics Committee of the Third People’s Hospital of Henan Province (Reference Number:2024-SZSYKY-016), which waived the requirement for informed patient consent based on the retrospective nature of the work.

## Results

### Clinical data

**Fig 1:**
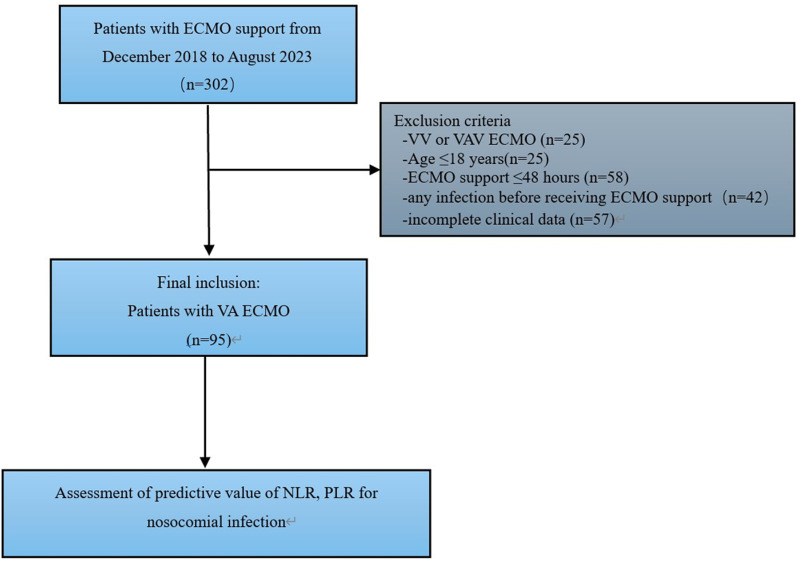
Study flow chart showing selection process of the study cohort. VA-ECMO: veno-arterial extracorporeal membrane oxygenation, VV: veno-venous, VAV: veno-arterial venous, NLR: neutrophil–to-lymphocyte ratio, PLR: platelet-to-lymphocyte ratio.

From December 2018 to August 2023, 302 consecutive patients received ECMO at the Department of Intensive Care Medicine of the Third People’s Hospital of Henan Province. Finally, 95 patients who received VA-ECMO for ≥48 hours were included (Fig 1). The clinical characteristics are summarized in Tables 1 and 2. There were 62 (65.26%) men with a mean age of 56.96 ± 15.29 years. The median duration of VA-ECMO treatment was 6.00 days (IQR 4.00–9.00). The median NLR and PLR levels were 13.71 (IQR 7.60–18.87) and 170.86 (IQR 104.71–283.87), respectively.

**Table 1:** Clinical characteristics of patients treated with VA-ECMO support.

Tertiles of NLR					
		1st tertile	2nd tertile	3rd tertile	
Characteristics	Total Cohort	0.94-9.77	9.78-16.34	16.35–49.23	P
N	95	31	32	32	
Male, n (%)	62 (65.26)	21 (67.74)	20 (62.50)	21 (65.63)	0.908
Age(years), mean ±SD	56.96 ± 15.29	55.10 ± 13.75	55.65 ± 15.64	60.09 ± 16.29	0.363
Combined diabetes, n (%)	30 (31.58)	6 (19.35)	11 (34.38)	13 (40.63)	0.176
Indications for VA-ECMO, n (%)					
Exacerbation of chronic heart failure	36(37.89)	11(11.58)	13(13.68)	12(12.63)	0.914
Acute myocardial infarction	12(12.63)	3(3.16)	3(3.16)	6(6.32)	0.581
Malignant arrhythmia	15(15.79)	6(6.32)	4(4.21)	5(5.26)	0.733
Pulmonary embolism	14(14.74)	2(2.11)	6(6.32)	6(6.32)	0.293
Cardiopulmonary resuscitation	10(10.53)	3(3.16)	3(3.16)	4(4.21)	
Other (Drug poisoning, etc.)	8(8.42)	2(2.11)	3(3.16)	3(3.16)	
Leukocyte(10^9^/L), median (IQR)	14.85 (10.93, 19.45)	14.38 (10.78, 18.79)	15.27 (11.37,21.59)	14.40 (10.48, 19.40)	0.804
Neutrophil (10^9^/L), median (IQR)	12.20 (9.22, 16.96)	11.67 (6.57, 14.46)	13.70 (10.10, 18.76)	13.47 (9.52, 17.71)	0.052
Lymphocyte (10^9^/L), median (IQR)	1.00 (0.61, 1.74)	2.19 (1.39, 4.81)	1.00 (0.75, 1.51)	0.55 (0.42, 0.90)	<0.001
Platelet (10^9^/L), mean ±SD	184.61 ± 74.54	188.10 ± 79.95	194.91 ± 74.99	170.94 ± 68.74	0.420
Hemoglobin (g/L), mean ±SD	110.76 ± 25.25	120.65 ± 25.29	107.53 ± 26.12	104.41` ± 21.97	0.024
Procalcitonin(ng/ml), median (IQR)	0.78 (0.17, 4.35)	0.25 (0.10, 4.35)	0.50 (0.12, 4.44)	1.48 (0.69, 7.33)	0.025
C-reactive protein(mg/L), median (IQR)	6.95 (0.95, 60.00)	3.10 (0.89, 67.80)	4.05 (0.60, 54.86)	18.06 (2.73, 55.61)	0.317
Albumin(g/L), median (IQR)	30.80 (27.30, 35.00)	30.00 (26.30, 35.90)	32.65 (28.73, 35.48)	29.70 (27.78, 33.50)	0.219
NLR	13.71 (7.60, 18.87)	5.59 (2.09, 7.60)	13.63 (12.17, 14.47)	22.91 (18.76, 27.92)	<0.001
the use of steroids during VA-ECMO initiations, n	0	0	0	0	
catecholamines infusions, n (%)	95(100)	31(32.63)	32(33.68)	32(33.68)	
Occurrence of nosocomial infection, n(%)	30(31.58)	4(12.90)	12(37.50)	14(43.75)	0.021
Duration of ECMO treatment (day),median (IQR)	6.00 (4.00, 9.00)	5.00 (3.00, 8.00)	5.00 (4.00, 11.75)	6.00 (3.25, 7.75)	0.520

Note: Values for continuous variables are expressed as mean ±standard deviation or median [interquartile ranges]; counts (percentages) are used for categorical variables.

**Table 2:** Clinical characteristics of patients treated with VA-ECMO support.

			Tertiles of PLR		
		**1st tertile**	**2nd tertile**	**3rd tertile**	
**Characteristics**	**Total Cohort**	15.14-131.60	131.61-241.43	241.44–711.76	P
N	95	31	32	32	
Male, n (%)	62 (65.26)	23 (74.19)	19(59.38)	20 (62.50)	0.430
Age(years), mean ±SD	56.96 ± 15.29	52.23 ± 15.34	55.78 ± 13.06	61.75 ± 16.47	0.074
Combined diabetes, n (%)	30(31.58)	4(12.90)	14(43.75)	12(37.50)	0.021
Indications for VA-ECMO, n (%)					
Exacerbation of chronic heart failure	36(37.89)	10(10.53)	12(12.63)	14(14.74)	0.642
Acute myocardial infarction	12(12.63)	3(3.16)	4(4.21)	5(5.26)	
Malignant arrhythmia	15(15.79)	5(5.26)	4(4.21)	6(6.32)	0.834
Pulmonary embolism	14(14.74)	3(3.16)	6(6.32)	5(5.26)	0.670
Cardiopulmonary resuscitation	10(10.53)	2(2.11)	3(3.16)	5(5.26)	
Other (Drug poisoning, etc.)	8(8.42)	3(3.16)	2(2.11)	3(3.16)	
Leukocyte(109/L), median (IQR)	14.85 (10.93, 19.45)	16.60 (12.33, 23.25)	17.35 (13.12, 20.44)	12.05 (8.46, 15.79)	<0.001
Neutrophil (10^9^/L), median (IQR)	12.20 (9.22, 16.96)	12.33 (9.22, 16.16)	15.43 (11.74, 18.30)	10.20 (7.40, 14.53)	0.001
Lymphocyte (10^9^/L), median (IQR)	1.00 (0.61, 1.74)	2.43 (1.50, 4.81)	1.13 (0.87, 1.44)	0.51 (0.39, 0.71)	<0.001
Platelet (10^9^/L), mean ±SD	184.61 ± 74.54	172.77 ± 81.03	198.00 ± 61.63	182.69 ± 79.79	0.404
Hemoglobin (g/L), mean ±SD	110.76 ± 25.25	120.13 ± 25.48	111.56 ± 24.23	100.88 ± 23.00	0.009
Procalcitonin(ng/ml), median (IQR)	0.78 (0.17, 4.35)	0.30 (0.10, 4.49)	0.77 (0.18, 2.08)	1.44 (0.40, 9.61)	0.069
C-reactive protein(mg/L), median (IQR)	6.95 (0.95, 60.00)	6.50 (0.95, 67.80)	3.22 (0.50, 34.47)	20.33 (2.38, 116.07)	0.043
Albumin(g/L), median (IQR)	30.80 (27.30, 35.00)	30.30 (26.90, 35.10)	32.85(29.93,35.48)	29.20 (26.95, 33.15)	0.119
PLR	170.86 (104.71, 283.87)	72.05 (36.88, 104.71)	166.93 (148.89, 203.27)	333.74 (283.47, 440.03)	<0.001
the use of steroids during VA-ECMO initiations, n	0	0	0	0	
catecholamines infusions, n (%)	95(100)	31(32.63)	32(33.68)	32(33.68)	
Occurrence of nosocomial infection, n(%)	30(31.58)	3(9.68)	9(28.13)	18(56.25)	<0.001
Duration of ECMO treatment (day),median (IQR)	6.00 (4.00, 9.00)	5.00 (3.00, 8.00)	6.00 (3.25, 10.75)	6.00 (4.00, 8.75)	0.348

Note: Values for continuous variables are expressed as mean ±standard deviation or median [interquartile ranges]; counts (percentages) are used for categorical variables.

### Nosocomial infection after VA-ECMO

30 out of 95 patients developed the nosocomial infection after VA-ECMO. There were 40 nosocomial infection events of 30 patients, including bloodstream infection in 12 (30%), respiratory infection in 20 (50%), urinary tract infection in 7 (17.5%), and surgical site infection in 1 (2.5%). A total of 43 strains of pathogens were detected in patients with postoperative VA-ECMO infection, which were mainly Gram-negative bacteria (30 strains, 69.77%), Gram-positive bacteria (10 strains, 23.25%) and fungi (3 strains, 6.98%). The majority of Gram-negative bacteria were Acinetobacter baumannii (13 strains, 30.23%) and Klebsiella pneumoniae (8 strains, 6.98%). The majority of Gram-positive cocci were Enterococcus spp (5 strains, 11.63%), coagulase-negative staphylococcus (3 strains, 6.98%). The main fungal infection was Candida albicans (2 strains, 4.65%). No virus was detected.

### Association of NLR levels with nosocomial infections in patients treated with VA-ECMO

**Fig 2:**
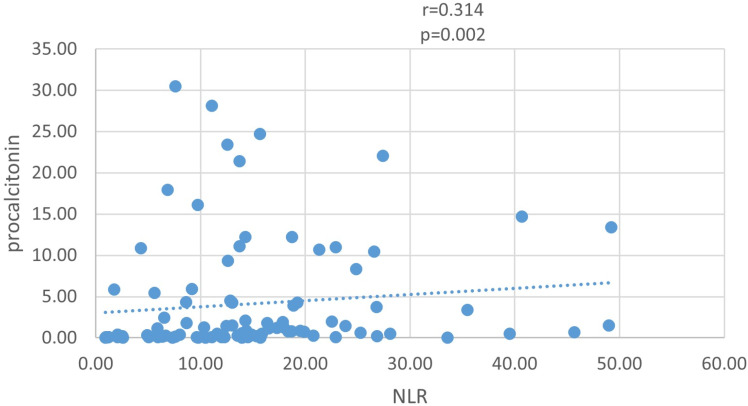
NLR level was positively correlated with procalcitonin (r = 0.314, p = 0.002).

The characteristics of patients stratified according to tertiles of NLR are summarized in Table 1 (first tertile: 0.94–9.77; second tertile: 9.78–16.34; third tertile: 16.35–49.23). Patients with higher levels of NLR showed higher rates of nosocomial infections, higher levels of procalcitonin, lower levels of Lymphocyte and lower levels of hemoglobin as shown in Table 1. We also analyzed the correlation between NLR levels and hemoglobin levels and procalcitonin levels. As shown in Fig 2, NLR levels were positively correlated with procalcitonin (r = 0.314, p = 0.002), and negatively correlated with hemoglobin (r = −0.297, p = 0.004) (S1 Fig). However, the correlation coefficient r was very low.

In the logistic regression model, NLR level was significantly associated with an increased risk of nosocomial infections in patients treated with VA-ECMO with a odds ratio [OR] of 4.858 (95% confidence interval [95%CI]1.864–12.663; P = 0.001) per ln-transformed (NLR) (Table 3). Compared to the first tertile of NLR, higher NLR level was a risk factor for the nosocomial infections in patients treated with VA-ECMO. After adjustment of traditional risk factors, including sex, age, hemoglobin, albumin, duration of VA-ECMO treatment, the ORs were 6.931(1.496–32.118) in the second tertile, 8.898(1.943–40.751) in the third tertile.

**Table 3:** Association of NLR levels with nosocomial infections in patients treated with VA-ECMO.

	Odds Ratio, 95% Confidence Interval and P value		
	Unadjusted	Model 1	Model 2
nosocomial infection after VA-ECMO	3.258(1.512-7.022)	3.140 (1.454-6.780)	4.858 (1.864-12.663)
per ln (NLR)	0.003	0.004	0.001
			
NLR tertiles			
1	1 (Reference)	1 (Reference)	1 (Reference)
2	4.050 (1.137-14.432)	4.043(1.128-14.488)	6.931 (1.496-32.118)
	0.031	0.032	0.013
3	5.250 (1.488-18.529)	4.910(1.378-17.498)	8.898 (1.943-40.751)
	0.01	0.014	0.005

Model 1 was adjusted for sex and age and sex was expressed as a dichotomous variable.

Model 2 was adjusted for the covariates in Model 1: hemoglobin, albumin, log-transformed duration of VA-ECMO treatment.

### Association of PLR levels with nosocomial infections in patients treated with VA-ECMO

**Fig 3:**
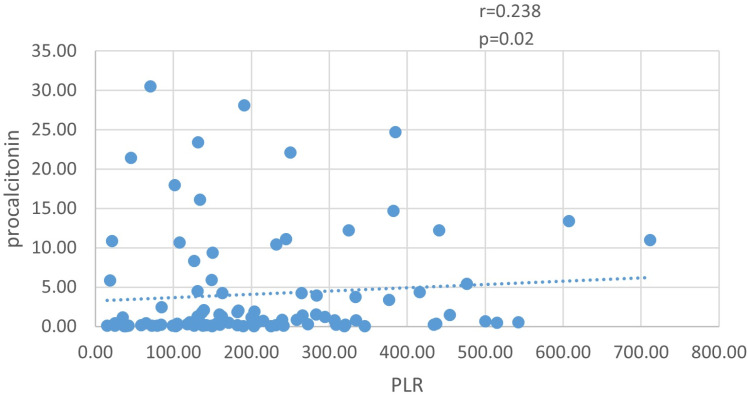
PLR level was positively correlated with procalcitonin (r = 0.238, p = 0.020).

Compared with lower levels of PLR, patients in the high group showed lower levels of lymphocyte, lower levels of hemoglobin and higher rates of nosocomial infections (Table 2) (first tertile: 15.14–131.60; second tertile: 131.61–241.43; third tertile: 241.44–711.76). As shown in Fig 3, PLR levels were positively correlated with procalcitonin (r = 0.238, p = 0.020), and negatively correlated with hemoglobin (r = −0.314, p = 0.002) (S2 Fig). However, the correlation coefficient r was very low.

In the logistic regression model, PLR was a risk factor for the nosocomial infections in patients treated with VA-ECMO outcome (odds ratio [OR] 5.478; 95% confidence interval [95%CI] 2.117–14.176; P < 0.001) after adjustment for sex, age, hemoglobin, albumin, duration of VA-ECMO treatment (Table 4). Using the first tertile of PLR levels as the reference, the ORs were 4.187 (95% CI, 0.898–19.534) in the second tertile, 13.500 (95% CI, 2.812–64.808) in the third tertile.

**Table 4:** Association of PLR levels with nosocomial infections in patients treated with VA-ECMO.

	Odds Ratio, 95% Confidence Interval and P value		
	Unadjusted	Model 1	Model 2
nosocomial infection after VA- ECMO	5.338(2.211-12.889)	5.198(2.131-12.680)	5.478 (2.117-14.176)
per ln (PLR)	<0.001	<0.001	<0.001
			
PLR tertiles			
1	1 (Reference)	1 (Reference)	1 (Reference)
2	3.652 (0.884-15.084)	3.626(0.870-15.116)	4.187 (0.898-19.534)
	0.073	0.077	0.068
3	12.000 (3.018-47.718)	11.457(2.811-46.698)	13.500 (2.812-64.808)
	<0.001	0.001	0.001

Model 1 was adjusted for sex and age and sex was expressed as a dichotomous variable.

Model 2 was adjusted for the covariates in Model 1: hemoglobin, albumin, log-transformed duration of VA-ECMO treatment.

### ROC analysis

**Fig 4:**
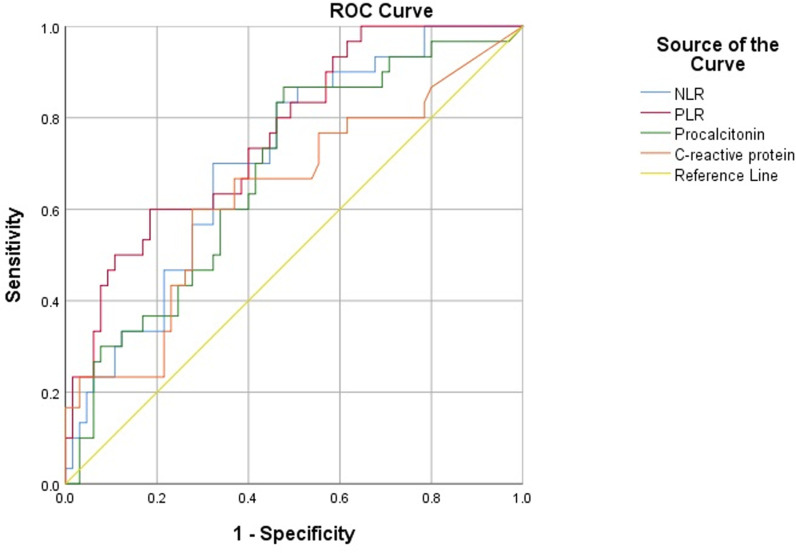
ROC curve showing NLR and PLR for predicting nosocomial infection in patients undergoing VA-ECMO.

ROC analysis of NLR revealed the area under the curve (AUC) of 0.710 [95% confidence interval (95%CI): 0.603–0.816; p = 0.001], the AUC of PLR was 0.763 [95%CI 0.664–0.863; p < 0.001]. The AUCs of procalcitonin and C-reactive protein were 0.681 [95%CI 0.568–0.793; p = 0.005] and 0.635 [95%CI 0.510–0.760; p = 0.035], respectively (Fig 4). The cut-off values of NLR, PLR, procalcitonin and C-reactive protein were 14.29 (sensitivity 70%, specificity 67.7%), 247.32 (sensitivity 60%, specificity 81.5%), 0.45 (sensitivity 86.7%, specificity 52.3%), 19.47 (sensitivity 60%, specificity 72.3%), respectively. NLR and PLR have more predictive value than procalcitonin and C-reactive protein for nosocomial infection treated with VA-ECMO.

## Discussion

ECMO is an essential form of life support for patients experiencing severe cardiac and/or respiratory failure [23]. Despite the advancements in ECMO as an extracorporeal life support technology, it still carries a high mortality rate and numerous potential complications. Due to long stays in intensive care units, direct vascular intubation, and other invasive procedures such as bronchoscopy, patients undergoing ECMO are at an elevated risk of acquiring nosocomial infections. Advanced age, presence of autoimmune comorbidities, and greater clinical severity have been identified as heightened risk factors for nosocomial infections during ECMO support [24]. Several studies have indicated that an extended duration of ECMO treatment was associated with an increased incidence of infection [25–27]. However, our analysis did not establish a link between longer ECMO duration and higher infection risk. This discrepancy may stem from the fact that NLR/PLR captured early inflammatory states prior to infection onset, and the median ECMO duration in our cohort was only 6 days (IQR 4–9), which might be insufficient to capture duration-dependent effects. A meta-analysis revealed that the prevalence of nosocomial infection ranged from 8.8% to 64.0%, with an incidence between 1.7 and 85.4 per 1000 ECMO days, and in-hospital mortality rate varying from 31.5% to 75.4% [7]. Consistent with this study, our study found that the rate of nosocomial infection was 31.58% in patients treated with VA-ECMO. The occurrence of nosocomial infections is correlated with increased mortality rates [28]. Therefore, early identification and reduction of risk factors for nosocomial infection play a pivotal role in influencing the prognosis of patients receiving ECMO therapy.

The initiation of ECMO induces a systemic inflammatory response, including the activation of endothelial cells, the complement system, platelets, and white blood cells, as well as a clotting cascade, which may result in increased morbidity and mortality [14]. The inflammatory response elicited by ECMO is a response to the blood’s exposure to the extracorporeal circulation [12]. Based on the current literature, systemic inflammation is recognized as a pivotal factor in the intricate pathophysiology of critical illness, and it is well documented that systemic inflammation is correlated with adverse outcome in patients receiving VA-ECMO therapy [14]. Peripheral blood leucocyte ratios, such as NLR and PLR, are useful infection biomarkers [29]. NLR reflects the balance between innate immune activation (neutrophils) and adaptive immune suppression (lymphopenia), both critical in infection pathogenesis. Neutrophilia is caused by de-margination of neutrophils, delayed apoptosis of neutrophils, and stimulation of stem cells by growing factors (G-CSF) during systemic inflammation. The mechanisms responsible for lymphopenia involve margination and redistribution of lymphocytes within the lymphatic system and marked accelerated apoptosis. The physiological immune response of circulating white blood cells to various stressful events as tissue injury, severe trauma, sepsis syndrome, is characterized by elevation of neutrophils and decline in lymphocyte counts [30]. PLR integrates the dual effects of platelet activation and lymphopenia. In addition to their role in hemostasis, platelets are a key mediator of inflammation during ECMO. The bound platelets within circulation have also been confirmed to be capable of facilitating the formation of leukocyte conjugates which was majorly combined with the monocytes and neutrophils, indicating that the bound platelets could influence the aberrant metabolism of leukocyte which further induce the inflammatory response [12].

In this study, we found that patients with higher levels of NLR and PLR showed higher rates of nosocomial infections(P < 0.05), we also found that patients with higher levels of NLR and PLR showed lower levels of Lymphocyte (P < 0.001) (Tables 1 and 2). We think that the rises of NLR and PLR in nosocomial infection in patients treated with VA-ECMO is mainly related to the decrease of lymphocytes. Lymphocytopenia is thought to be a direct impact of heightened serum cortisol and catecholamines in systemic stress responses [31,32]. Most patients supported by ECMO are in a state of high stress, which easily leads to systemic inflammatory response and decreased immunity. Previous researches have indicated that low lymphocyte count is correlated with adverse prognoses in patients with diverse cardiac pathologies, including acute coronary syndromes [31]. Therefore, the presence of lymphocytopenia may increase the susceptibility to infection, which is a well-known factor contributing to mortality and functional decline. This fact may be regarded as circumstantial evidence indicating a biological connection [31,33].

High neutrophil count and low lymphocyte count are frequently observed in acute inflammatory responses [31]. NLR has been extensively studied in various diseases, especially cardiovascular pathology [34]. Sebastian et al. [35] found that NLR is independently associated with in-hospital mortality in patients undergoing VA-ECMO. It has also been reported that NLR is significantly increased in pneumonia compared with healthy people, suggesting that it can be used as a predictor of the presence of pneumonia [36]. In this retrospective cohort study, we found that higher NLR level was still a risk factor for the nosocomial infections in patients treated with VA-ECMO after multivariable adjustment. In reference to the first tertile of NLR, ORs were 6.931 (95% CI, 1.496–32.118) for the second tertile, 8.898 (95% CI, 1.943–40.751) for the third tertile (Table 3). Therefore, higher NLR were associated with an increased risk of nosocomial infections in patients undergoing VA-ECMO.

Platelets play a pivotal role in mediating inflammation during ECMO [14]. Studies have shown that PLR can provide more comprehensive predictive information about inflammation and aggregation pathways than platelets or lymphocytes alone [37]. Previous studies have found that PLR is a new marker for poor prognosis of cardiovascular disease [38]. Kundi et al. [39] established a significant association between high PLR and mortality in patients diagnosed with acute pulmonary embolism. However, they did not evaluate PLR as a marker showing any relationship between the risk of developing nosocomial infections. In this study, we found that high PLR is associated with nosocomial infections in patients treated with VA-ECMO, and it is one of the risk factors.

In our study, ROC analysis showed that the AUC of NLR and PLR to predict nosocomial infections in patients treated with VA-ECMO were 0.710 and 0.763, respectively. The AUCs of procalcitonin and C-reactive protein were 0.681 and 0.635, respectively. Our ROC analysis demonstrates that PLR (AUC = 0.763) outperforms NLR (AUC = 0.710), PCT (AUC = 0.681), and CRP (AUC = 0.635) in predicting nosocomial infections among VA-ECMO patients, suggesting its potential as a more reliable biomarker in this specific population. The superior specificity of PLR (81.5% vs 67.7% for NLR) at the optimal cutoff of 247.32 makes it particularly valuable for ruling in infections, while NLR’s higher sensitivity (70%) may be useful for initial screening. some studies have proven NLR to be at least a moderate predictor of bacteremia, with AUROCs ranging from 0.7 to 0.77 [29,40,41]. Another study reported that the optimal cutoff value of NLR for diagnosing sepsis was 7.97, with a sensitivity of 64.26%, a specificity of 80.16%, and an AUC of 0.74 (p < 0.001)[42]. Notably, the NLR cutoff of 14.29 is substantially higher than values reported in non-ECMO populations, likely reflecting the baseline systemic inflammatory state induced by ECMO circuitry. The relatively lower specificity of NLR (67.7%) may be attributed to ECMO-related neutrophil activation independent of infection [14]. While PCT demonstrated the highest sensitivity (86.7%), its poor specificity (52.3%) limits its standalone diagnostic value. We found that NLR and PLR, especially PLR, have more predictive value than procalcitonin and C-reactive protein for nosocomial infection in patients treated with VA-ECMO.

We observed expected correlations between NLR/PLR and procalcitonin, supporting the biological plausibility of our findings. While the negative NLR/PLR-hemoglobin correlations may reflect anemia-associated inflammation, hemoglobin levels are influenced by multifactorial processes (e.g., bleeding, transfusions), limiting the specificity of this association in ECMO patients. Their clinical utility requires further validation.

To the best of our knowledge, there is no study to explore the association between NLR, PLR and nosocomial infection in patients treated with VA-ECMO. In this cohort study, we found that high NLR and PLR were associated with an increased risk of nosocomial infection in patients treated with VA-ECMO. The present study has several limitations. First, this was a single-center retrospective study, and large-sample multicenter prospective clinical studies are still needed for further validation. Second, the current study lacked a non-ECMO control group, preventing direct comparison of the specificity of NLR. Future studies should prospectively evaluate NLR in parallel ECMO and non-ECMO cohorts to establish population-specific cut-offs. Third, due to the relatively small sample size, we were unable to perform subgroup analyses stratified by specific underlying etiologies (e.g., acute myocardial infarction). This may have obscured potential differences in the predictive value of NLR across distinct patient populations. Future multicenter studies with larger cohorts should validate our findings in specific disease subgroups. Fourth, our study focused on NLR and PLR within 24 hours of ECMO initiation, which while clinically actionable for early decision-making, does not capture potential later inflammatory dynamics. Future prospective studies should incorporate serial measurements to evaluate temporal patterns. Fifth, while catecholamine use was universal in our cohort, the lack of granular dosing data precludes analysis of its potential modulating effects on inflammatory markers. Future prospective studies should incorporate detailed vasoactive medication records

## Conclusion

In conclusion, in this study, we demonstrated an association between NLR, PLR and nosocomial infection in patients treated with VA-ECMO. High NLR and PLR were associated with an increased risk of nosocomial infection in patients treated with VA-ECMO.

## Supporting information

S1 Fig NLR level was negatively correlated with hemoglobin (r = −0.297, p = 0.004). (TIF)

S2 Fig PLR level was negatively correlated with hemoglobin (r = −0.314, p = 0.002). (TIF)
