# Supplementary figures and images for: Neutrophil-to-lymphocyte ratio and platelet-to-lymphocyte ratio as potential predictors of nosocomial infection in patients undergoing veno-arterial extracorporeal membrane oxygenation: A cohort study
Source: PLoS One. 2025 Jun 3;20(6):e0325316. doi: 10.1371/journal.pone.0325316 (PMC12133176; doi:10.1371/journal.pone.0325316)

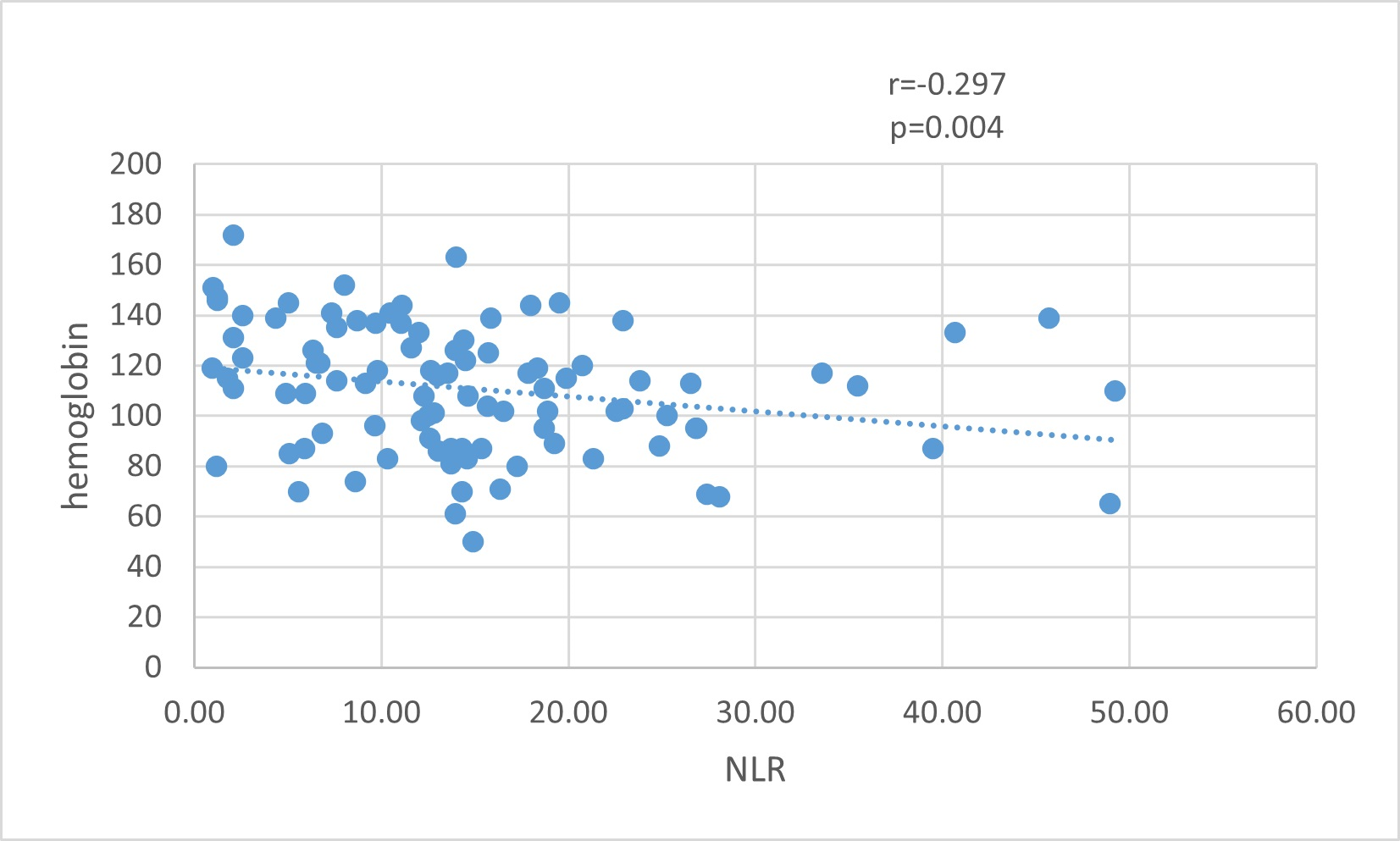

Supplement: S1 Fig — (TIF) [file pone.0325316.s001.tif]

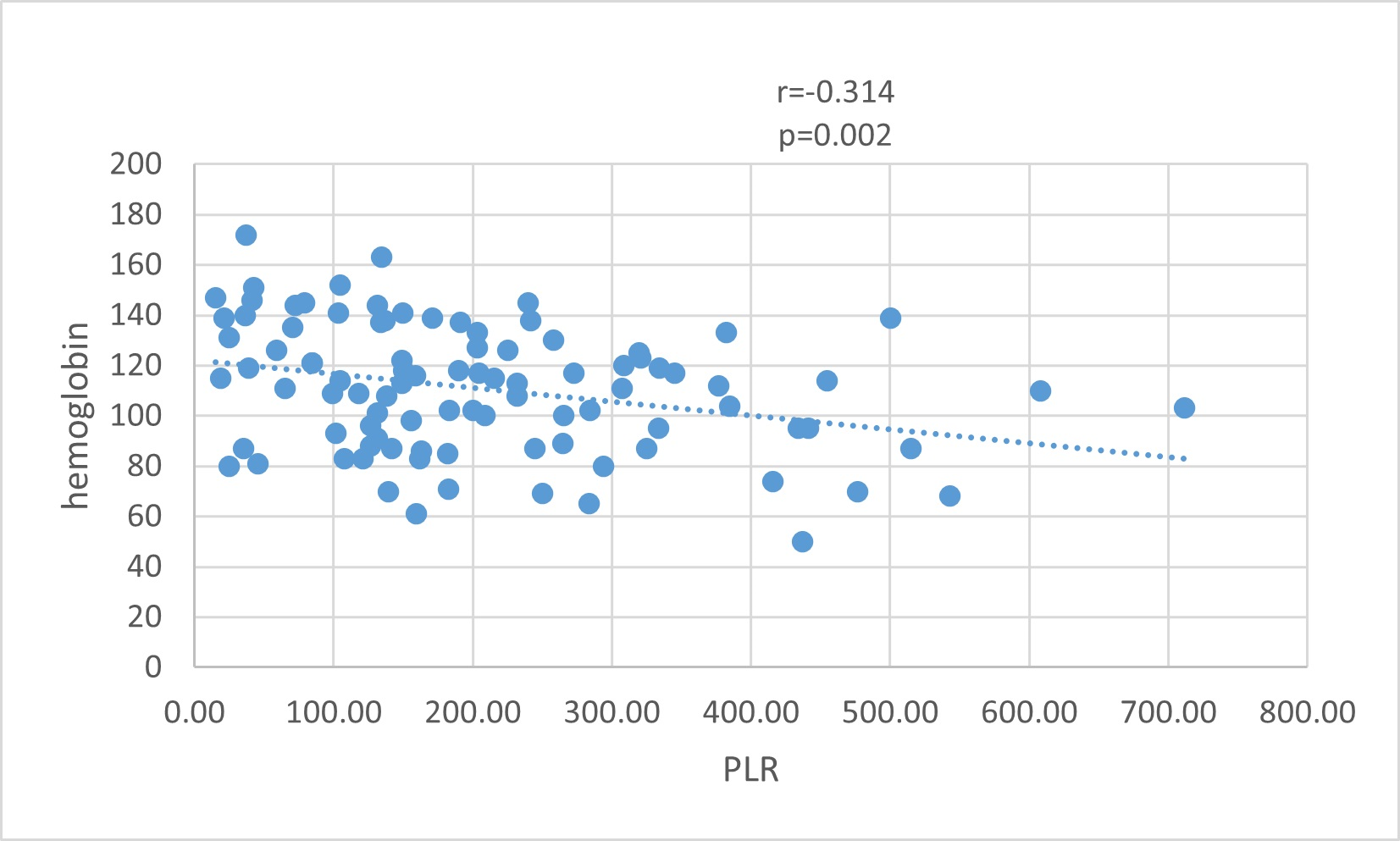

Supplement: S2 Fig — (TIF) [file pone.0325316.s002.tif]
